# High prevalence and low cure rate of tuberculosis among patients with HIV in Xinjiang, China

**DOI:** 10.1186/s12879-016-2152-4

**Published:** 2017-01-05

**Authors:** Rena Maimaiti, Yuexin Zhang, Kejun Pan, Peierdun Mijiti, Maimaitiali Wubili, Maimaitijiang Musa, Rune Andersson

**Affiliations:** 1 Department of Prevention and Health Care, The First Affiliated Hospital of Xinjiang Medical University, Urumqi, China; 2 Department of Infection Diseases, The First Affiliated Hospital of Xinjiang Medical University, Urumqi, Xinjiang China; 3 Department of Epidemiology and Biostatistics, Xinjiang Medical University, Urumqi, China; 4 The Clinic of Liudaowan Hospital of Urumqi, Urumqi, Xinjiang China; 5 Department of Infectious Diseases, Institute of Biomedicine, Sahlgrenska Academy at Gothenburg University, Göteborg, Sweden

**Keywords:** HIV, Tuberculosis, Isoniazid prophylaxis, Treatment outcome

## Abstract

**Background:**

Tuberculosis and HIV co-epidemics are problems in many parts of the world. Xinjiang is a high tuberculosis and HIV prevalence area in China. We aimed to investigate the prevalence and cure rate of tuberculosis among HIV positive patients in Xinjiang.

**Methods:**

In a retrospective study between 2006 and 2011, clinical and laboratory data on 333 patients with HIV and tuberculosis were compared to 2668 patients with HIV only. There were 31 HIV positive patients with no data on tuberculosis.

**Results:**

The prevalence of tuberculosis co-infection among the HIV positive patients was 11% (95% CI 10–12%), significantly higher than the national figure in China of 2%. In most cases HIV was diagnosed late, with advanced immunodeficiency. The use of isoniazid preventive therapy was only 57.9% in patients without tuberculosis who fulfilled the criteria for receiving this prevention. The cure rate one year after diagnosis was 69.2%, which was lower than the officially reported 91.4% in all tuberculosis cases in Xinjiang in 2011. The hazard of not surviving over the five years was significantly higher in patients with HIV + tuberculosis compared to HIV only after adjusting for sex and Intravenous drug use with HR = 1.84 (95% CI 1.43-2.35; *p* < 0.0001).

**Conclusions:**

The prevalence of tuberculosis among HIV positive patients in Xinjiang was higher than in China taken as a whole, and HIV was diagnosed late, with underuse of isoniazid preventive therapy. The low cure rate and reduced survival can be due to late diagnosis of HIV and no testing for antibiotic resistance, together with insufficient control of adherence to the treatment regimens for tuberculosis and HIV.

## Background

*Mycobacterium tuberculosis* (TB) and human immune deficiency virus (HIV) infections are major public health problems in many parts of the world, particularly in low and middle-income countries [1, 2]. In 2013, an estimated 9.0 million people developed TB and 1.5 million died from the disease. Of these, about 1.2 million (14%) had a HIV co-infection [3]. China has the world’s second largest number of tuberculosis cases after India (12 and 26% of global cases, respectively) in 2012 [4]. WHO estimated the prevalence of TB (including HIV + TB) in China 2012 to be 1,400,000 (1,200,000–1,600,000). The incidence rate of new TB infections was 99 (86–113)/100,000 [4].

Several studies have indicated that TB co-infection increases the risk of HIV progression and death, particularly in persons with untreated HIV [5].

HIV infection is associated with immunosuppression, which in turn increases the risk of latent TB infection developing into active TB [6, 7].

After contact with tuberculosis bacteria, patients with HIV have 20 times the normal incidence of TB [8]. This risk can be reduced by isoniazid preventive therapy (IPT), with benefits for both the individual and for the further spread of TB, and it is generally accepted that the lifetime risk of TB reactivation in the HIV positive group can be reduced from about 30 to 4%[9, 10].

HIV positive patients with TB and low CD4^+^ T-lymphocyte counts (<100 cells/mm^3^), more often present with atypical chest X-rays and negative acid-fast bacilla (AFB) sputum smears, compared to HIV-negative patients [11].

Xinjiang Uygur Autonomous Region is a province in the Northwestern part of China, with 21,815,815 inhabitants in 2011. The incidence of TB in Xinjiang was 2003–2008 estimated at 463 cases per 100,000 persons per year [12]. The information on the prevalence of multidrug-resistant (MDR) and extensively drug-resistant (XDR TB) remains scant in the region, due to TB drug susceptibility testing (DST) not being implemented as a routine test at most hospitals. It was performed in combination with a clinical study in Urumqi from 2009 to 2011, and indicated a prevalence of MDR TB of 13.2% [13].

One study from January 2007 to June 2010, including 11 counties in Xinjiang, reported that the HIV screening rate was 79.1% in the TB patients’ group, and the rate of positive HIV was 2.2% among those tested. TB was tested for in 85.3% of the HIV positive patients, of whom 10.8% were diagnosed with TB [14].

At screening for HIV among patients with tuberculosis in the 15 counties in Xinjiang 2007, the prevalence was 1.2% [15]. At the TB hospital, Urumqi, all patients with TB were tested for HIV between 2009 and 2012. 6.2% of the TB patients were co-infected with HIV, with stable prevalence rates over time.

The availability of HIV testing in Xinjiang was not the same the whole time period and many times offered in projects directed towards high risk groups. In Xinjiang the HIV infection prevalence in the drug users was 21.4% from one study 2010 [16].

## Objectives

The objectives of the study were to investigate the prevention, prevalence, and cure rate of tuberculosis among HIV-positive patients in Xinjiang, China.

## Methods

A cross-sectional retrospective study was carried out between November 2006 and December 2011, and identified 3032 HIV-positive patients at the HIV clinics of three city hospitals in Xinjiang, China. The patients were treated in Urumqi (729), Yili (1861) and Kuqa (442). The total populations in the three cities were respectively 3.11 million (2011), 515,000 (2011) and 475,000 (2010).

Doctors and nurses specializing in infectious diseases at these hospitals were responsible for the collection of data. Register forms were used to collect data on demographic factors and clinical features.

The 2001 revised diagnostic criteria for the diagnosis and treatment of pulmonary tuberculosis were used [17]: 1. Positive culture of *Mycobacterium tuberculosis* from sputum, tissue, blood, liver, spleen, lymph nodes, biopsy or pleural effusion. 2. Persistent high fever (>38.5 °C) more than two weeks, or night sweats, or more than 10% weight loss (3–6 months),or weakness. 3. Positive tuberculin test (PPD), where the scleroma was moderate (10–19 mm) or intense (>20 mm), or with blister, local necrosis, lymphangitis). 4. Chest X-ray image showing changes typical of tuberculosis. Patients fulfilling the first criterion, or two of the others, were diagnosed with TB.

All the patients, irrespective of whether they had signs and symptoms of chest infection, were screened for pulmonary TB by chest X-ray, and then by examination of sputum for AFB using standard techniques. Blood samples were collected periodically every 6 months and tests included a CD4 cell count. Routine blood tests included red blood cells, white blood cells, platelet count and hemoglobin. HIV viral loads were not routinely analyzed.

Patients with CD4+ T lymphocyte count <350 /μl, or CD4+ T lymphocyte count >350 /μl together with one of the following symptoms were eligible for anti-retroviral treatment: enlarged lymph nodes,persistent fever for several months, night sweats, more than 10% weight loss, diarrhea, generalized herpes infection, oral *Candida albicans* infection.

The first line antiretroviral treatment (ART), given was two nucleoside reverse transcriptase inhibitors (NRTIs) plus a non-nucleoside reverse transcriptase inhibitor (NNRTIs) as combination therapy. The available first line drug combinations were: zidovudine (AZT) + lamivudine (3TC) + nevirapine (NVP), AZT + 3TC+ efavirenz (EFV) or stavudine (D4T) + 3TC+ NVP. The second line treatments were tenofovir (TDF) + 3TC + lopinavir (LPV) or TDF + 3TC + EFV. The drugs were supplied by the state free of charge.

In China, the National TB Control Program (NTP) is implemented directly through a network of TB centers and TB hospitals, and is also used at HIV clinics [18]. Tuberculosis drug treatment is generally given according to a standard scheme: H (isoniazid),R (rifampicin), Z (pyrazinamid), E (ethambutol) for 3 months, then reduced to isoniazid and rifampicin for an additional 3 months. Some patients (like patients with TB meningitis, or chest X-ray without improvement) were given extended treatment for 9 months or one year. The HIV positive patients treated at the HIV clinics were followed-up in the actual study. For the HIV negative patients treated at the TB centers and TB hospitals, we have no data on adherence to treatment or follow-up programs, and only reports of case fatalities. We have no information about whether the patients in our study had been treated at TB centers previously.

HIV positive patients with CD4 < 100/μl were recommended IPT. In addition, patients with CD4 < 250/μl together with weight loss, lymph node enlargement or PPD where the scleroma was 5–10 mm, were recommended IPT.

In this study, no patient underwent the TB resistance test, because it was not available at the general hospitals in Xinjiang. The study from between 2009 and 2011 on the prevalence of MDR-TB [13] did not include the patients in this study.

During the treatment, the patients were monitored for CD4 cell count, levels of liver enzymes, chest X-ray in cases with lung symptoms like cough or sputum.

### Statistical methods

For comparison of proportions between groups we used Chi-square test. The differences in hazard between groups were analyzed by Cox proportional hazard analysis. Survival curves based on Kaplan-Meier estimates are presented. *P*-values < 0.05 were regarded statistically significant.

We analyzed the cure rate one year from the diagnosis of TB. We defined a TB cure as patients surviving the treatment period, having a negative sputum smear if previously the smear was positive, no remaining TB- related symptoms, and an improvement in chest or extrapulmonary X-ray findings, and no relapse after the end of TB treatment, relapse was defined as having a positive culture 30 days after the last treatment date [19]. Patients who stopped treatment before full term and patients lost to follow-up were regarded as treatment failures. Patients who transferred to other clinics, and surviving patients followed for less than one year, were excluded from the analysis of cure rate.

## Results

**Fig. 1:**
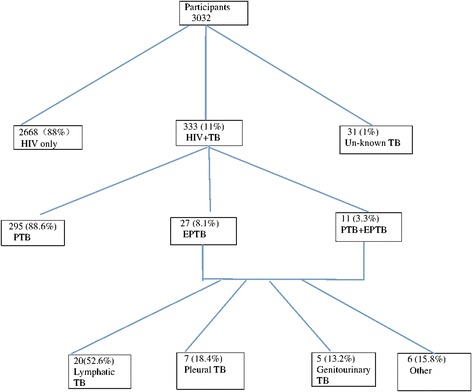
Flow chart detailing recruitment and TB presentation

The flow chart describing the included patients in the study is shown in Fig. 1.

### Sociodemography

The demographic data on the patients with HIV + TB and HIV only are shown in Table 1.

**Table 1:** Sociodemographic and clinical data

		HIV + TB *N* = 333	HIV only *N* = 2668
Sex	Men	242	1492
	Women	91	1176
Age	<29	3	85
	30-39	105	917
	40-49	184	1316
	>50	41	381
Ethnic	Uygur	287	2273
	Han	30	308
	Hui	10	109
	Other	6	9
Routes of infection	Blood	3	26
	IVDU	194	1053
	Homosex	1	38
	Sex	94	1190
	MTCT	0	1
	Unknown	23	231
	No data	18	160
CD4	<100	122	492
	100-249	138	1083
	>250	71	1118
	No data	2	6
Opportunistic infections	Oral candida infection	33	49
	PCP	11	6
	Oral hairy leukoplakia	2	16
	KS	4	14
	Generalized herpes infection	12	48
	Others	9	27

*Abbrevations*: *IVDU* (intravenous drug users), *MTCT* (mother-to- child transmission), *PCP* (Pneumocystis Jiroveci pneumonia), *KS* (Kaposis Sarkoma)

The prevalence of TB among the HIV positive patients was 333/3032 (11%), it was higher in men than in women (14% vs 7%, *p* < 0.05).

For the patients with extra-pulmonary TB (EPTB) the mean age was 39.2 years (median 38, range 27–52).

The main route of the HIV infection among patients with HIV + TB was Intravenous Drug Use (IVDU) at 58.3% (95% CI 53–63.6), which was significantly higher than for patients with HIV only, at 38.9% (95% CI 37.1-40.7); *p* <0.05.

Of the patients with HIV + TB, 72.7% (95% CI 68–77.4) were men, which was significantly higher than for patients with HIV only where the figure was 55.3% (95% CI 53.4-57.2).

Hepatitis B surface antigen (HBsAg) was positive in 21 (6%) of all 333patients with HIV + TB, with 14/21 (67%) being HBV-DNA positive. Hepatitis C antibodies were found in 83 (25%) of the 333 patients, and 33/83 (40%) were HCV-RNA positive.

Among the 1247 IVDUs 38% were HBsAg positive and 40% were HCV- RNA positive. Data on treatment for hepatitis were not registered, but treatments were not free and it is likely most patients were untreated.

#### Clinical symptoms

The symptoms among patients with HIV + TB and HIV without TB are shown in Table 2. As shown, fever, cough, sputum production and night sweats were more common in the HIV + TB patients.

**Table 2:** Symptoms among patients with HIV + TB, and HIV only

	HIV/PTB *N* = 295 *n* (%; 95% CI)	HIV/EPTB *N* = 38 *n* (%; 95% CI)	HIV + TB *N* = 333 *n* (%; 95% CI)	HIV only *N* = 2668 *n* (%; 95% CI)
Fever	125 (42.4; 36.7-48.1)	12 (32; 17.1-46.9)	137 (41.1; 35.8-46.6)^*^	392 (14.7; 13.3-16.1)^*^
Cough	142 (48.1;42.4-53.8)	7 (18; 5.8-30.2)	149 (44.7; 39.4-50)^*^	472 (17.7; 16.3-19.1)^*^
Productive cough with sputum	116 (39.3;33.8-44.8)	5 (13; 2.2-23.8)	121 (36.3;31.2-41.4)^*^	389 (14.6; 13.2-16)^*^
Dyspnea	28 (9.5;6.2-12.8)	4 (11; 1–21)	32 (9.6; 6.5-12.7)^*^	107 (4.0; 3.3-4.7)^*^
Sternalgia	41 (13.9;10–17.8)	3 (8; 0–16.6)	44 (13.2; 9.7-16.7)^*^	104 (3.9; 3.2-4.6)^*^
Night sweats	116 (39.3;33.8-44.8)	11 (29; 14.5-43.5)	127 (38.1; 32.8-43.4)^*^	344 (12.9; 11.6-14.2)^*^
Diarrhea	22 (7.5;4.5-10.5)	4 (11; 1–21)	27 (8; 5–11)	253 (9.5; 8.4-10.6)
Nausea	27 (9.0;5.7-12.3)	2 (6.0; 0–13.4)	28 (8.4; 5.4-11.4)	240 (9.0; 8–10)
Projectile vomiting	3 (1.0;0–2.1)	2 (6.0; 0–13.4)	3 (1.0; 0–2.1)	27 (1.0; 0.6-1.4)
Headache	38 (12.9;9.2-16.6)	2 (6.0; 0–13.4)	40 (12.0; 8.5-15.5)	312 (11.7; 10.5-12.9)
Blurry vision	16 (5.4;2.9-7.9)	1 (3; 0–8.5)	17 (5.1; 2.7-7.5)	179 (6.7; 5.8-7.6)
Rash	12 (4.1;2.1-6.1)	2 (6.0; 0–13.4)	14 (4.2; 2.2-6.2)	115 (4.3; 3.5-5.1)
Lymphadenectasis	34 (11.5;7.8-15.2)	11 (29; 14.5-43.5)	45 (13.5; 9.8-17.2)^*^	160 (6.0; 5–7)^*^

*P < 0.05

The average CD4 count at the time of HIV diagnosis was 169/μl in patients with HIV + TB, (median 146/μl; range 2–931), compared to 230/μl (median 223, range 1–1668) in the patients without TB. Out of the patients with HIV + TB 51.1% (95% CI 45.8-56.4) had CD4 counts of less than 150, which was significantly more common than in patients with HIV only, at 35.2% (95% CI 33.4-37).

Thirty-eight patients (11.4%) had EPTB, of which 28 (74%) were men. The largest proportion of EPTB cases had tubercular lymphadenitis (53%), followed by pleural tuberculosis (18%).

#### Treatment of tuberculosis and HIV

Of the 333 patients with TB, 292 (87.7%) received treatment for TB. Six surviving patients had less than one year of follow-up, 7 (2.4%) were lost to follow up, and 4 (1.4%) were transferred to other clinics. Among the remaining 275 patients, 256 (93.1%) started the TB treatment before the ART. But we have no any details regarding reasons for stopping treatment and for being lost during follow-up period.

#### Isoniazid preventive therapy

Among the 1161 patients without TB who fulfilled the criteria for IPT, it was given to 57.9% during the study period. Patients with intravenous drug use (IVDU) had lower rates of IPT 51.4% (95% CI 47.7-55.1) than non-IVDU patients, where 63.1% (95% CI 59.4-66.8) received the treatment. Men had a lower rate of IPT at 53.7% (95% CI 50–57.4), than women, at 64.5% (95% CI 61–68). Of the IVDU patients, 93.9% were men. No statistically significant difference was seen between ethnic groups, see Table 3.

**Table 3:** Isoniazid preventive therapy (IPT) for patients fulfilling the criteria for IPT

	fulfilling the criteria for IPT (*n* = 1161) *N*	IPT (*n* = 672) *N* (%; 95% CI)
All	1161	672
Men (*n* = 1734)	713	383 (53.7;50–57.4)
Women (*n* = 1298)	448	289 (64.5;61–68)
Uygur (*n* = 2560)	992	571 (57.6;53.9-61.3)
Han (*n* = 338)	124	72 (58.1;54.4-61.8)
Other (*n* = 134)	45	29 (64;60.5-67.5)
IVDU (*n* = 1247)	519	267 (51.4;47.7-55.1)
Non-IVDU (*n* = 1785)	642	405 (63.1;59.4-66.8)
Ongoing follow-up (*n* = 2107)	670	527 (78.7; 75.7-81.7%)
Stop-ART (*n* = 367)	125	42 (33.6;30.1-37.1)
Death (*n* = 426)	280	70 (25;21.7-28.3)
Lost follow-up (*n* = 106)	77	27 (35; 24–46)
Transferred (*n* = 25)	9	6 (67)

#### Cure rate and survival

In our study the cure rate one year after diagnosis of TB could be evaluated for 234 patients and was 162/234 (69.2%). Twenty-three other patients (9.8%) had improved. No improvement in symptoms was seen among 18 patients (7.7%) and 31 (13.2%) had died. The causes of death were unfortunately not registered.

**Fig. 2:**
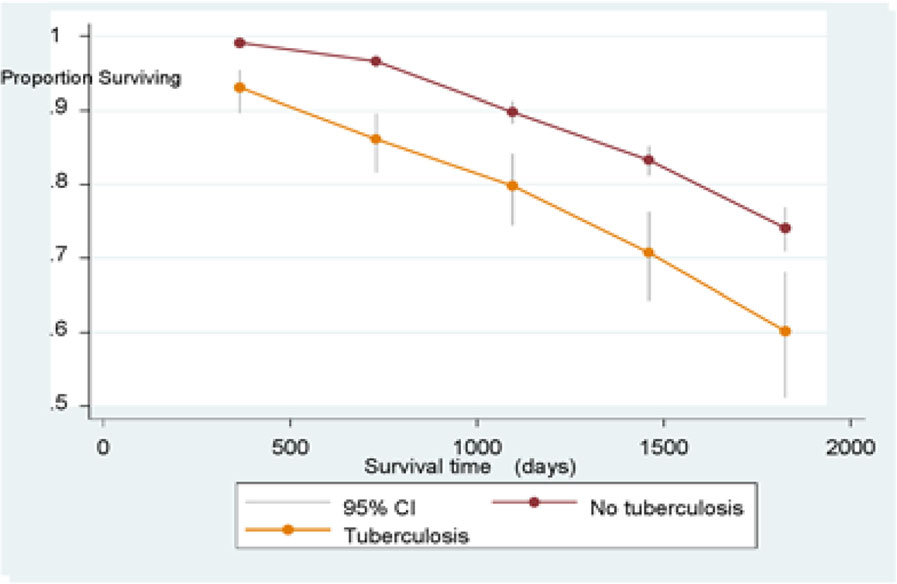
Survival time in 333 patients with HIV + tuberculosis and in 2668 with HIV only

The survival time of HIV + TB patients was significantly shorter than in the patients with HIV only (Fig. 2). We note that reduced survival time among the patients with TB was marked during the first year, with no additional reduction of survival the following years (Fig. 2). The hazard of not surviving over the five years was significantly higher in patients with HIV + TB compared to HIV only after adjusting for sex and IVDU with HR = 1.84 (95% CI 1.43-2.35; *p* < 0.0001).

**Fig. 3:**
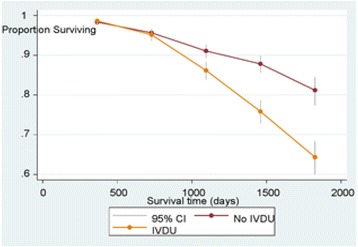
Survival time in 1247 patients with intravenous drug use (IVDU) compared to 1353 patients with no IVDU. (For 432 patients data were missing)

For the patients with IVDU there was no significantly different survival the first year. However, during the following years, this group had reduced survival rates year by year starting from the second year (Fig. 3).

Among the 333 patients with HIV + TB data on other opportunistic infections were available for 275 patients. The surviving 203 patients had a median time of follow-up on 39 months. Out of them 21 (10.3%; 95% CI 6.2-14.4%) had one or more previous or actual opportunistic infections (10 oral candida infection,6 generalized herpes infection, 2 *Pneumocystis jiroveci* pneumonia (PCP), 2 oral hairy leukoplakia, 1 Kaposi sarcoma.). Among 72 patients who had died, 16 had oral candida, 3 generalized herpes infection, 5 PCP, 2 Kaposi sarcoma. The rate of opportunistic infections in dead patients was 26/72 (36% (95% CI 24-47%), statistically significantly higher than among the surviving patients.

## Discussion

The prevalence of TB co-infection in the HIV positive patients in Xinjiang was 11% (95% CI:10–12%), which was significantly higher than the national figure in China of 2% [4] in 2012 and also tended to be higher than in a study from Tanzania on 340 women coming to voluntary counselling and testing (VCT) center for HIV test with a figure of 5.3%(95% CI:0–10.6%) [20]. In this study, we found that the higher prevalence of HIV + TB in men on 14% compared to 7% in women (*p* < 0.05). This could be due to the fact that 67.5% of the men and only 5.9% of the women were IVDU. TB/HIV co-infection was more prevalent in men also in a Tanzanian study [20], but in Tanzania there were not many IVDUs, but alcohol abuse was common among men.

Out of the patients in our study, 87.7% of patients co-infected with HIV and TB started treatment for both HIV and TB. This is a better figure than for China as a whole, where it is reported that only 23% of TB patients were tested for HIV in 2011 with 4715 (2%) being HIV positive. Of these, only 1677 (36%) received ART [21].

More than half of the patients with HIV + TB (51.1%) had CD4 counts of less than 150, which was significantly more common than in patients with HIV only, at 35.2%. It indicated a late diagnosis of HIV among the patients with TB.

Among the 1161 patients fulfilling the criteria for isoniazid prophylaxis, only 57.9% received the prophylaxis. Men had lower rate for prophylaxis treatment than women in our study. This could due to the fact that most of IVDUs were men, for whom preventive treatment is more difficult.

Extrapulmonar tuberculosis (EPTB) was seen among 11.4% (95% CI:8.1-14.4) of patients in our study, which is similar to the global figure of 13% on all TB cases reported by WHO [22], but low compared to what is seen in study with 757 patients with HIV in Ethiopia with 25% (95% CI:19.7-30.3) [23]. We found that in our cases the most common types of EPTB were lymphadenitis (53%) and pleural TB (18%). Peto et al. published a wide series of EPT cases from the USA and revealed that lymphatic (40%) and pleural (19.8%) types of EPTB were the most frequent, and meningeal involvement was found in 5.4% of their cases [24].

The cure rate of TB in our study was 162/234 (69.2%) (95% CI 66.2–72.2%), lower than the officially reported 91.4% for all cases of TB in Xinjiang 2011 [25], but similar as results of a study from Zimbabwe with 225 adult TB patients with a figure of 70% (95% CI 50–90) [26]. The cure rate for EPTB was 34.2%. There is no previously reported data from Xinjiang, but results are similar to those of a study from Taiwan of 28.7% [27]. Mortality among patients with EPTB was 36.8% in our study,compared to 14.7% in a study from the United States [28]. Low TB cure rates among patients with HIV can be due to late HIV diagnosis with advanced immunodeficiency and no testing of TB resistance. Insufficient control of adherence to treatment regimens makes it impossible to evaluate if this can be a cause of the low cure rates. Both the treatment of TB and HIV need a high level of adherence to be successful.

According to WHO estimates, approximately 1 million new TB cases and 54,000 MDR-TB cases in 2013 [29] occur in China, Xinjiang is one of the areas in China with the highest prevalence [13]_._ The incidence of drug resistance is higher in the north, west, and central areas of China, and lower in south and east China [30–32].

However, testing for resistance was not performed in Xinjiang during the studied period, which blocked optimal treatment and control of TB.

Rapid and timely detection of TB cases and strengthened capacity to diagnose cases of drug-resistant TB are thus global priorities for TB care and control [7]. The Gene Xpert MTB/RIF test on sputum can in about 2 h detect 73% of culture positive acid fast bacilla (AFB) smear- negative TB cases from one specimen, and 90% after three specimens in the study by Boehme et al. in study from Peru, Azerbaijan, South Africa, and India [33]. In a study in Ethiopia Gene Xpert had a sensitivity on culture- positive smear-negative TB cases of 57.4% with one sputum sample and 67.6% with two samples [34]. That and similar tests could allow for more rapid detection of tuberculosis and drug resistance outside reference centers, and cut delays in diagnosis, without the need to build large numbers of laboratories equipped to meet advanced biosafety [33] requirements. The problem is however the high cost.

Treatment discontinuation is considered as one of the risk factors for MDR-TB [35–38]. Although TB control and prevention in the Centre of Disease Control and prevention (CDC) system have professional follow-up systems, the patients from the hospitals are sometimes missed at transfer from hospitals to local TB dispensaries, and will thus not complete their follow-up treatment and get a high risk of drug resistance [30].

In this study the survival time of the IVDU patients was shorter than of non-IVDU patients. One important reason for low survival among HIV + IVDU patients can be the high proportion of patients with untreated hepatitis B and C. Of the patients with IVDU 27% were HBsAg positive and 40% were HCV-RNA positive. There was no free treatment for hepatitis B or C in Xinjiang [39].

There are very limited economic resources in Xinjiang compared to developed cities like Beijing, Shanghai and Guangzhou. Although the Chinese government in recent years has implemented a new healthcare system, there is not enough money for patients in the whole country to receive good medical treatment from the onset of the disease.

There is also limited skill in medical treatment in most of the rural hospitals. In addition, the patients’ living conditions are often poor, with people crowding in the same living spaces to reduce costs of heating.

In the case of TB symptoms, many patients did not pay attention to them, because of lack of money. Although the TB drug are free for tuberculosis patients, they must afford the cost for auxiliary treatment, including drugs for reducing adverse reactions [40]. They have to pay for travel costs, tests and hospital care, which can discourage them, and may in turn lead to delayed diagnosis and treatment, with increased risk of spreading TB.

According to the results of a national general survey of tuberculosis between 2001 and 2010 in China, only 47% of patients with pulmonary tuberculosis went to the hospitals, and knowledge of TB prevention and control was only 57% [41].

A beneficial effect of immediate antiretroviral therapy was evident for reductions in rates of TB, Kaposi’s sarcoma, and malignant lymphomas, meanwhile, the significant benefit in the immediate antiretroviral therapy in patients with HIV infection regardless of CD4+ count [42]. Hence, economic assistance should be available to ensure that TB patients with economic hardship can complete treatment, thus reducing the emergence of drug-resistant TB [40].

### Limitations

This study was carried out in HIV clinics from 3 hospitals in Xinjiang. The data is dependent on the quality of the patient records, and we did not include CDC clinics, and private hospitals. For these reasons the results might not reflect the overall TB situation in this region. We lack data on the treatment outcomes of patients who were transferred. We have no data on whether patients were living in cities or rural areas. We also lack data on the causes of death.

## Conclusions

The low cure rate of TB and reduced 5- year survival in the HIV positive patients could be caused by late diagnosis of HIV, no availability of TB resistance tests, together with a lack of documented high levels of adherence to HIV and TB treatment regimens.

Hence, economic assistance should be available to ensure that TB patients with economic hardship can complete treatment, thus reducing the emergence of drug-resistant TB.

## Abbreviations

AFB: Acid-fast bacilla
ART: Antiretroviral treatment
AZT: Zidovudine
CDC: Centre of disease control and prevention
DST: Drug susceptibility testing
D4T: Stavudine
EFV: Efavirenz
ELISA: Enzyme-linked immunosorbent assay
EPTB: Extra-pulmonary tuberculosis
HIV: Human immune deficiency virus
IPT: Isoniazid preventive therapy
IVDU: Intravenous drug use
LAM: Lipoarbinomannan
LPV: Lopinavir
MDR: Multidrug-resistant
NNRTIs: Non-nucleoside reverse transcriptase inhibitor
NRTIs: Nucleoside reverse transcriptase inhibitors
NTP: National TB Control Program
NVP: Nevirapine
PCP: *Pneumocystis jiroveci* pneumonia
PPD: Purified protein derivative skin test
TB: Tuberculosis
TDF: Tenofovir
XDR: Extensively drug-resistant
3TC: Lamivudine
VCT: Voluntary counselling and testing
